# Penetrating stab injuries in Iceland: a whole-nation study on incidence and outcome in patients hospitalized for penetrating stab injuries

**DOI:** 10.1186/s13049-018-0582-2

**Published:** 2019-01-23

**Authors:** Una Johannesdottir, Gudrun Maria Jonsdottir, Bergros K. Johannesdottir, Alexandra Aldis Heimisdottir, Elias Eythorsson, Tomas Gudbjartsson, Brynjolfur Mogensen

**Affiliations:** 1 Department of Cardiothoracic Surgery, Landspitali University Hospital, Reykjavik, Iceland; 2 Department of Anesthesiology and Critical Care, Yale New Haven Hospital, New Haven, CT USA; 3 Department of Surgery, Haukeland University Hospital, Bergen, Norway; 4 Faculty of Medicine, University of Iceland, Reykjavik, Iceland; 5 Department of Internal Medicine, Landspitali University Hospital, Reykjavik, Iceland; 6 Department of Emergency Medicine, Landspitali University Hospital, Reykjavik, Iceland

**Keywords:** Stabbing injury, Trauma, Penetrating, Treatment, Mortality, Outcome

## Abstract

**Background:**

Studies on penetrating injuries in Europe are scarce and often represent data from single institutions. The aim of this study was to describe the incidence and demographic features of patients hospitalized for stab injury in a whole nation.

**Materials and methods:**

This was a retrospective nationwide population-based study on all consecutive adult patients who were hospitalized in Iceland following knife and machete-related injuries, 2000–2015. Age-standardized incidence was calculated and Injury Severity Score (ISS) was used to assess severity of injury.

**Results:**

Altogether, 73 patients (mean age 32.6 years, 90.4% males) were admitted during the 16-year study period, giving an age-standardized incidence of 1.54/100,000 inhabitants. The incidence did not vary significantly during the study period (*P* = 0.826). Most cases were assaults (95.9%) occurring at home or in public streets, and involved the chest (*n* = 32), abdomen (*n* = 26), upper limbs (n = 26), head/neck/face (*n* = 21), lower limbs (*n* = 10), and the back (*n* = 6). Median ISS was 9, with 14 patients (19.2%) having severe injuries (defined as ISS > 15). The median length of hospital stay was 2 days (range 0–53). Forty-seven patients (64.4%) underwent surgery and 26 of them (35.6%) required admission to an intensive care unit (ICU), all with ISS scores above 15. Three patients did not survive for 30 days (4.1%); all of them had severe injuries (ISS 17, 25, and 75).

**Conclusion:**

Stab injuries that require hospital admission are rare in Iceland, and their incidence has remained relatively stable. One in every five patients sustained severe injuries, two-thirds of whom were treated with surgical interventions, and roughly one-third required ICU care. Although some patients were severely injured with high injury scores, their 30-day mortality was still low in comparison to other studies.

## Background

Trauma is one of the leading causes of death in all age groups in most developed countries [1, 2], and globally an estimated five million people die every year from trauma—injuries that are in most cases preventable and non-random [2]. Most of these deaths are from blunt trauma related to motor vehicle accidents, but in some parts of the world including the United States, South Africa, and Australia [3–5], penetrating trauma from either gunshot wounds or stab injuries is a major healthcare problem [3–7]. In South Africa, gunshot wounds are by far the most common form of penetrating injury but in the United States and Australia stab injuries predominate [3, 4, 8]. Penetrating injuries are less common in Northern Europe and are therefore not a frequent cause of major injury or death [9–12]. As in the United States and Australia, stab injuries predominate over gunshot wounds in Northern Europe, especially in the Nordic countries. In Sweden and Finland, for example, stab injuries are six or seven times as common as gunshot wounds [10, 12].

Penetrating stab injuries, especially deep injuries involving internal organs and large vessels in the chest or abdomen, can lead to infections, shock, exsanguination, and death [4]. Early diagnosis and treatment is therefore of paramount importance using the assessment tools that are available such as focused assessment with sonography, computed tomography, and local wound exploration [13–17]. In earlier reports, the mortality rate has ranged from 2% in a low-volume centre in Norway [11] to 15% from severe trauma at a major Australian urban trauma centre [18]. Some of these differences can be explained by differences in the severity of injuries, and in what types of injuries and injured body parts were included in the studies.

There have been very few European studies on penetrating trauma published, especially from urban areas. Furthermore, there have been no studies representing whole populations, with most reports representing single trauma centres in larger cities [3, 9, 18, 19].

The aim of this study was to determine the incidence, demographic features, and outcome of penetrating stab injuries in Iceland.

## Methods

### Design and population

This was a retrospective cohort study involving all the patients who were hospitalized in Iceland following a penetrating stab injury some time between 1 January 2000 and 31 December 2015. The mean size of the population over the 16-year study period was 309,360 and at the end of December 2015, Iceland had a population of 332,529 [20].

### Trauma care in Iceland

Iceland is a sparsely populated country with primary trauma care provided in small hospitals in each quarter of the country. The only tertiary trauma centre, Landspitali University Hospital, is located in the capital city (Reykjavik) where about 213,000 people live within 6.3 min of ambulance transport time to the hospital [21, 22]. There is another trauma centre located in the north of Iceland, Akureyri, which serves a population of around 20,000–25,000 in the northern and eastern parts of Iceland. Other hospitals in Iceland are smaller and have limited resources for treating critically injured patients, as they have no intensive care units (ICUs). Thus, patients are taken to either Landspitali Hospital or Akureyri Hospital by ambulance, helicopter, or aeroplane for further evaluation and treatment.

### Study population and data sources

Included in the study were all patients ≥ 18 years of age who sustained a penetrating stabbing injury (with a knife or machete) and were admitted to hospital. Patients who were treated in the emergency department but discharged without admission were excluded from the study. Patients who died on site or in the emergency department before being admitted to hospital (*n* = 15) were included only for calculation of total mortality. Data on these patients were obtained from the National Cause of Death Registry in Iceland and compared to the autopsy databases of the Medical Examiner Office of Iceland and the Department of Pathology at Landspitali Hospital, where all autopsies in Iceland are performed.

All the hospitals in Iceland maintain a database of all admissions, and they are categorized using the International Classification of Diseases Version 10. Data collected from all regional hospitals and databases were cross-checked to make sure that all cases were included in the study. These databases included a separate ICU database and diagnosis and operation registries at Landspitali Hospital and all regional hospitals.

For each patient, the following data were collected from medical records: age, sex, date of injury, type of injury (assault, self-inflicted injury, or accident), anatomical site of injury, whether intoxicated with alcohol when injured, surgical procedures, ICU admission, blood transfusions, length of stay (both in the ICU and total hospital stay (in days)), and 30-day and hospital mortality.

### Severity of injury

Injury classification was done by calculation of anatomical and physiological scores using the Abbreviated Injury Scale (AIS), the Injury Severity Score (ISS), the New Injury Severity Score (NISS) and the Revised Trauma Score (RTS). Of these, AIS, ISS, and NISS are all anatomical scoring systems used to rate the severity of each injury to the body whereas the RTS takes into account the physiological parameters such as Glasgow coma scale, respiratory rate, and systolic blood pressure on first medical contact.

### Statistical analysis

Data were entered into a Microsoft Excel spreadsheet. All statistical analyses were performed using R Statistics version 3.4.4. Descriptive analyses were performed for both continuous and categorical variables. Normally distributed variables were summarized as mean with 95% confidence intervals (CI) and compared using Student’s t-test. Variables with skewed distributions were summarized with their median and interquartile range (IQR) and compared using Wilcoxon’s rank sum test. Rates were calculated using age and gender-specific population estimates from Statistics Iceland and they were standardized using the World Health Organization’s World Standard Population. Age standardization was accomplished using the direct method and confidence intervals were calculated assuming the gamma distribution. The incidence of stab injuries by study periods was estimated using Poisson regression.

The study was approved by the Icelandic National Bioethics Committee and the Data Protection Authority. As individual patients were not identified, individual consent for participation in the study was not obtained from them.

## Results

### Number of cases and incidence

**Fig. 1:**
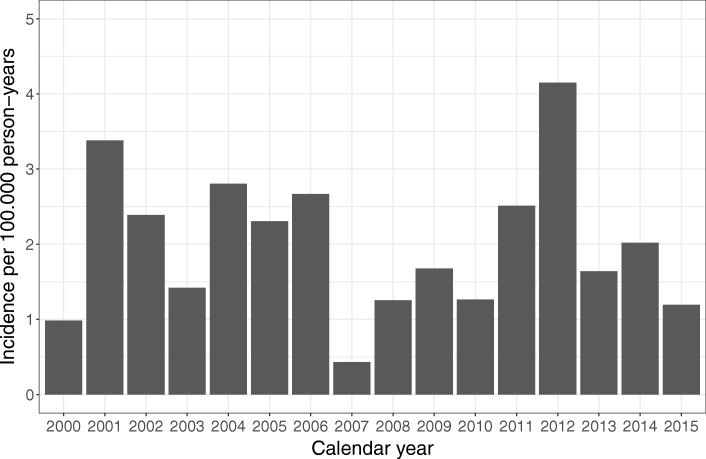
Incidence of penetrating stab injuries during the 16 – year study period

During the 16-year study period, a total of 88 patients had penetrating stab wounds but 15 of them died on site or in the emergency department before being admitted to hospital, eight due to homicide, six due to suicide, and one for unknown reasons. Out of the 73 patients a total of 23 cases were admitted 2000–2004, 19 cases 2005–2009 and 31 cases 2010–2015. For the whole 16-year study period the age-standardized incidence of penetrating stab injuries in individuals 18 years or more was 1.54 per 100,000 inhabitants (95% CI 1.21–1.94). The incidence of penetrating stabbing injuries did not vary significantly during the study period, incidence rate ratio 1.00 (95% CI 0.58–1.73, *P* = 0.826). The incidence is shown in Fig. 1. The mean age of the 73 patients who were admitted alive was 32.6 years, with an age range of 18–68 years, and 59 (80.8%) of them were less than 45 years old (Table 1). Sixty-six of the patients (90.4%) were male, giving a male to female ratio of 9:1. Seventy patients (95.9%) were treated at Landspitali Hospital and three in Akureyri Hospital. The characteristics of these patients are given in Table 1.

**Table 1:** Characteristics of stab-injured patients

	n (%)	Range
Number of patients	73	
Mean age, years	32.6	18–68
Number of males	66 (90.4)	
Anatomic site of injury		
Head/face/neck	21 (17.4)	
Thorax	32 (26.4)	
Abdomen	26 (21.5)	
Back	6 (5.0)	
Upper limbs	26 (21.5)	
Lower limbs/pelvis	10 (8.3)	
Number of patients operated on	47 (64.4)	
Number of patients requiring ICU admission	26 (35.6)	
Median ISS	9	1–75
Median NISS	9	1–75
Median total hospital stay, days	2	0–53
Median length of ICU admission, days	1	1–38
30-day mortality	3 (4.1)	
Median ISS for patients who died	39	

*ISS* Injury Severity Score, *NISS* New Injury Severity Score, *ICU* intensive care unit

### Anatomical distribution, cause, and location of injury

The anatomical distribution of injuries is shown in Table 1. Injuries to the chest, abdomen, and upper limbs were most common (26.4, 21.5, and 21.5%, respectively), with 28 patients (38.4%) sustaining injuries in more than one body region.

The most common cause of injury was assault, which had occurred in 70 cases (95.9%), followed by three cases of self-inflicted injuries (4.1%). In two out of these three cases, the patients were female. The most common cause of injury caused by assault was domestic violence (54.8%), followed by violence in public streets (31.5%), in bars and nightclubs (8.2%), and in the workplace (4.1%). In one case, the cause was unknown.

### Treatment

Of 73 patients who were admitted alive, 47 (64.4%) underwent a total of 60 surgical procedures. Table 2 shows a list of the procedures; soft tissue repairs (*n* = 23) and chest tube insertion (*n* = 19) were the most common interventions, followed by 12 laparotomies or laparoscopies and six emergency thoracotomies and sternotomies. Of the 73 patients, 26 (35.6%) required admission to the ICU (Table 1).

**Table 2:** Surgical interventions

	n (%)
Total interventions	60
Thoracic:	
- Tube insertion	19 (31.7)
- Emergency thoracotomy	3 (5.0)
- Sternotomy	3 (5.0)
Abdominal:	
- Laparoscopy	3 (5.0)
- Laparotomy	9 (15.0)
Other:	
- Vascular surgery	4 (6.7)
- Tendon/muscle/nerve repair	19 (31.7)

### Severity of injury and length of stay

Median ISS and NISS of all patients admitted were 9 (IQR 2–11) and 9 (IQR 3–11), respectively. Patients admitted to the ICU had significantly higher ISS and NISS scores (median 14.5 (IQR 6–18.5) and 14.5 (IQR 6–23.8), respectively), but 14 patients (19.2%) had severe injuries, with an ISS or NISS over 15. The median length of hospital stay was 2 days (mean 4.4 days, IQR 1–4, range 0–53) and the median length of ICU stay was one day (mean 3.6 days, IQR 1–1.75, range 1–38).

### Outcome

Three of the 73 patients died within 30 days of admission, giving a 30-day mortality rate of 4.1%, and the in-hospital mortality rate was the same. Two of the patients who died had sustained injuries to more than one anatomical region, with an ISS of 17 and 75, and the third patient had a severe injury to the thorax only (ISS 25). These three patients all underwent emergency surgery, with one of them succumbing intraoperatively due to haemorrhagic shock. The other two died 11 and 15 days postoperatively from complications related to the initial injury.

## Discussion

This population-based study covered all knife and machete-related penetrating stab injuries that required hospitalization in Iceland over a recent 16-year period. The incidence of stab injuries requiring hospitalization in Iceland was very low (1.54 per 100,000 inhabitants), which is in line with other Nordic countries such as Finland (0.9 per 100,000 inhabitants) [10], but far lower than the much higher incidence reported in Australia (390 per 100,000 inhabitants) [23]. Furthermore, the incidence of stab injury requiring hospitalization in Iceland was relatively stable over time which is in line with recent studies from high-volume centres in Australia, but also Belgium, Sweden, and Finland [10, 24–26]. Generally,there has been a feeling of increased incidence of stab injuries in western societies, possibly as a result of media attention [9], but contrary to this belief, recent studies have shown that the overall incidence of such injuries does not appear to be increasing [3, 18, 24, 26, 27]. In fact, many studies have shown a decrease in incidence [18, 23].

Thirty-day mortality in the present study was very low (4.1%), but our cohort represents a small population compared to previously reported series in the literature [3, 24, 25, 27, 28]. This indicates that advanced trauma care can be provided for these patients in a small geographically isolated community such as Iceland, where the incidence of penetrating stab injury is very low. In Iceland, the trauma centre at Landspitali Hospital is run by physicians trained in emergency medicine, and trauma team activation triggers a well-defined triage guideline and protocols. The surgical staff on call is notified almost immediately about the trauma from ambulance paramedics, allowing them time to arrive at the emergency department when a trauma patient is on the way. Furthermore, all supervising physicians in Iceland are certified in advanced trauma life support.

Almost all cases in this study were assaults on men in the early decades of adult life. This is in line with previous studies, where penetrating stab injuries have mainly been observed in young men in the second and third decades of life [7, 18, 24, 25, 27–29]. A noticeable majority of cases in our study involved domestic violence, which contrasts with overseas studies where most of the injuries happen out of doors in public locations [18, 28]. This difference from our study can be explained by the much warmer climate in these areas, so people spend more time outdoors. Previous studies have shown that alcohol and drugs are commonly involved in penetrating stab injuries [5, 7, 10, 25–27], and many of the patients involved are unemployed [26] and belong to ethnic minorities [25, 27]. Concurrent consumption of alcohol was common in the current study, but other demographic features such as unemployment status and ethnicity were not reported.

In the present study, the body regions most commonly affected were the chest and abdomen, which is in line with other studies [18, 27, 30]. A high percentage of patients underwent surgical interventions in the study, in contrast to many overseas studies, which have usually involved high-volume centres where the approach of “watchful-waiting” or selective non-operative management is more commonly advocated. This includes studies from the United States and Australia where 55% [8] and 60–79% [27] of stab wounds to the anterior abdomen, respectively, have been treated non-operatively [30]. Of the surgical interventions in our study, acute laparotomy and laparoscopy were the most common procedures. This is not surprising, as 21.8% of the injuries were to the abdominal region. Overseas studies have shown that at certain lower-volume centres similar to ours, such as Norway [11], for example, operative management is the more common choice. Patient care has evolved in recent years, with most high-volume trauma centres favouring a more conservative, non-operative approach [8, 25, 30, 31]. This indicates that choosing a selective non-operative approach requires experience, and that treatment strategies are frequently based on the experience of the attending physician involved at any particular time.

It has been reported that a non-operative approach can delay the diagnosis of organ injuries. Thus, extreme caution is recommended when choosing this approach at small/low-volume trauma centres where expertise regarding penetrating trauma may be less than at larger centres [31]. Still, at both low- and high-volume centres the most common operations are laparotomies and laparoscopies, and even though the indications for very recently introduced surgical procedures such as emergency-department thoracotomy (EDT) are still being debated, the outcome of EDT for penetrating injuries to the heart appears to be proving more encouraging as the years pass [32]. This is reflected in our own experience at Landspitali where we have treated several patients with EDT, including some following penetrating knife injury [33].

The main strength of this study was its nationwide design, representing a well-defined study population with a modern nationalized healthcare system. Furthermore, it covered a relatively long period of time (16 years) and was based on reliable, detailed data extracted from medical records from all primary and tertiary care centres, from autopsy databases, and from the Cause of Death Registry at Statistics Iceland. The main limitation of this study stems from its relatively small number of cases and its retrospective design. It only covered stab injuries in adult patients, and the age-standardized incidence does not therefore represent all ages. Furthermore, only hospitalized patients were analyzed, and not those who died on the scene or before reaching hospital. Finally, the Trauma and Injury Severity Score, which determines the probability of survival using numbers from the ISS/NISS and RTS, could not be reported as documentation of vital signs — in particular, the respiratory rate and the Glasgow coma scale score — was often missing on first medical contact during the first few years of the study period. The documentation has, however, improved significantly in recent years in Iceland and will be more reliable in future studies.

## Conclusions

This population-based study, covering a whole nation study and using all adult cases registered, has shown that penetrating stab injuries are rare in Iceland and that the incidence is not increasing. A large proportion of the patients needed surgical interventions, and their 30-day mortality was low despite having moderate-to-severe injuries.

## Abbreviations

AIS: Abbreviated injury scale
CI: Confidence interval
EDT: Emergency-department thoracotomy
ICU: Intensive care unit
ISS: Injury severity score
NISS: New injury severity score
RTS: Revised trauma score
